# Deep learning enables nanoscale X-ray 3D imaging with limited data

**DOI:** 10.1038/s41377-023-01198-z

**Published:** 2023-06-27

**Authors:** Chonghang Zhao, Hanfei Yan

**Affiliations:** National Synchrotron Light Source II, Brookhaven National Laboratory, Upton, NY 11973 USA

**Keywords:** Optics and photonics, Physics

## Abstract

Deep neural network can greatly improve tomography reconstruction with limited data. A recent effort of combining ptycho-tomography model with the 3D U-net demonstrated a significant reduction in both the number of projections and computation time, and showed its potential for integrated circuit imaging that requires high-resolution and fast measurement speed.

X-ray tomography is a non-destructive imaging technique that provides three-dimensional (3D) structural information about an object. It has many applications in various scientific fields. The technique involves taking a series of projection images as the sample rotates, and then using a mathematical algorithm to compute the volumetric reconstruction. The quality of the reconstruction depends on both the data and the computation method. To achieve a given resolution, the number of projections must meet the Crowther criterion, which means that hundreds to thousands of projections are required under optimal conditions^1^. Regarding tomography algorithms, they can be mainly divided into two categories: direct methods that utilize back-projection or the connection between Radon and Fourier transforms^2–4^, and model-based methods that iteratively solve an optimization problem^5,6^. Direct methods require less computation but reconstructions are more susceptible to artifacts with poor-quality data. In contrast, iterative methods are more robust but are less efficient.

In some cases, it is not possible to meet the data requirements due to concerns about radiation dose or geometrical constraints. For example, the tomography of integrated circuits (ICs) is limited by its plate-like geometry, which restricts the collection of projection images to a limited angular range. This leads to a well-known missing edge problem in tomography. In addition, it is desirable to take as few projections as possible to speed up the measurement and cover a large area. Achieving high-quality reconstruction with fast measurement and light computation has been a focus of research for a long time.

The recent emergence of deep neural networks (DNNs) has opened up new opportunities to tackle this challenge. DNNs enable machines to learn complex and implicit knowledge, allowing them to provide artificial intelligence (AI). By harnessing the power of DNNs, researchers have been able to achieve nearly perfect reconstruction with incomplete datasets, a task that was previously difficult or impossible with conventional methods^7–9^.

DNNs can be used in many ways in tomography. They can act as a black box to replace the iterative solver and directly map the measured data to the target image^9–12^. They can also serve as a post-image processing tool to improve the image after the reconstruction^7,8^. Additionally, they can perform sinogram inpainting to fill in missing information in the measurement or regularize a solution in a model-based approach to preserve desired properties^13–15^.

A recent paper by Z. Wu et al. demonstrates another successful marriage of DNNs and tomography^16^. The team from MIT and Argonne National Laboratory successfully reconstructed an IC sample using only 21 projections in a range of 140 degrees. Their proposed method, named RAPID, greatly reduces the required amount of data and speeds up computation time by 140 times while retaining fine details of the object and achieving a similar quality to that obtained from a full dataset with 349 projections.

RAPID differs from existing efforts in two aspects (See Fig. 1). First, it employs a multi-slice propagation method to model the measurement, which accounts for the diffraction effect inside the object^17,18^. This potentially removes the limitation on object thickness imposed by the depth of view and allows for the achievement of spatial resolution better than that of the optical system. Using a Fresnel Zone plate with an outermost zone width of 50 nm, the team was able to achieve a voxel resolution of 14 nm.

**Fig. 1:**
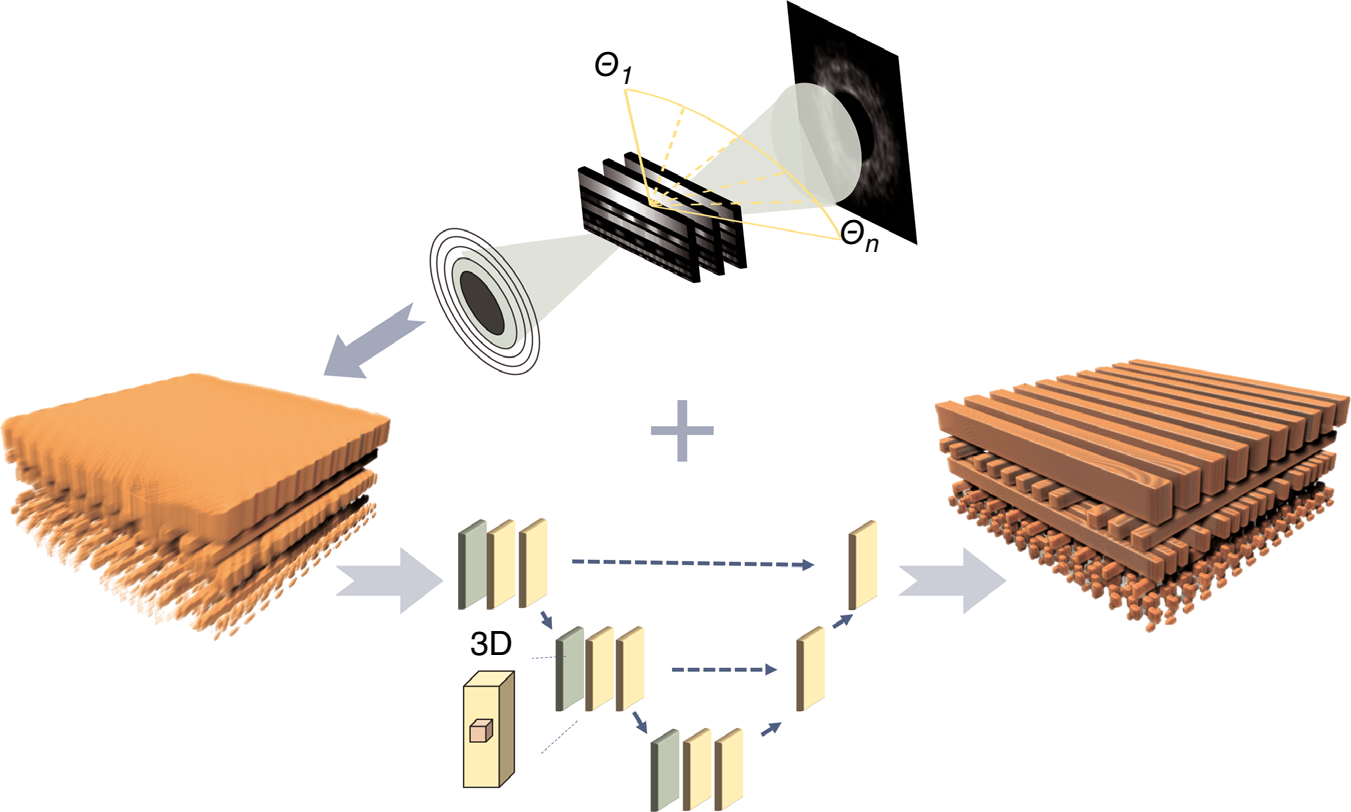
An x-ray nanoprobe illuminates an IC sample at different angles, where multi-slice model is employed to account for the diffraction effect. With limited number of projections, a poor-quality approximant was first obtained using ptycho-tomography algorithm, and then fed to the 3D U-net to produce a nearly-perfect tomogram. The network was trained on the reconstruction with the full dataset acquired from the same sample

Second, RAPID implements a 3D U-net with atrous convolutions for larger receptive fields, allowing the network to capture 3D features more efficiently. The combination of a 3D physical model and a 3D network makes it possible to dramatically reduce both the number of projections and computation time for a tomogram. It also eliminates the normalization issue between slices sometimes seen with 2D methods.

In RAPID, supervised learning is used. The dataset acquired from the IC sample was split into two parts: one for training and one for testing. A high-resolution reconstruction was generated using the conventional two-step approach of first reconstructing the 2D projection images and then performing tomographic reconstruction. This reconstruction was used as the ground truth for training. Various metrics were used to evaluate the performance of the method, and RAPID consistently outperformed conventional filtered back-propagation (FBP) and simultaneous algebraic reconstruction techniques (SART).

The proposed strategy is to train the network on a subset of the sample where sufficient measurements have been conducted and good results have been achieved with conventional methods. The network can then be applied to the rest of the sample where a very sparse dataset has been collected to significantly speed up the process. One drawback is that new training may be required for a different sample, but transfer learning may reduce the effort if the features are similar.

With the power of DNNs, which enforce solutions to meet expectations based on prior knowledge, visually appealing results can be obtained from incomplete or sometimes “trash” datasets. This is the case with RAPID and other AI-enabled algorithms. These methods can greatly reduce the effort required to measure similar samples while still maintaining quality. However, because the network is trained on prior knowledge, the solution is biased toward that by design. One caveat is that if there is a new feature that was not seen during training, the network may ignore or misinterpret it. This is not an issue if we do not expect any surprises from a sample, such as in a screening application. However, in scientific research where unknown features are of interest, this raises several open questions.

Is the solution unique? Could there be another visually appealing solution that is equally probable? Can uncertainty be assigned to different locations of the object to indicate which portions are more “guessed” by AI? Unlike conventional methods where a “bad” result is easily recognizable, an AI-enabled tool can generate an output that always looks “good”. To what degree can we trust it? Appropriate metrics may need to be defined to describe the confidence level.
